# Patterns of care for brachytherapy in Japan

**DOI:** 10.1093/jrr/rrad099

**Published:** 2023-12-26

**Authors:** Hitoshi Ikushima, Noriko Ii, Shin-ei Noda, Koji Masui, Naoya Murakami, Ken Yoshida, Miho Watanabe, Shinnji Kawamura, Toru Kojima, Yoshihito Nomoto, Takafumi Toita, Tatsuya Ohno, Hideyuki Sakurai, Hiroshi Onishi

**Affiliations:** Department of Therapeutic Radiology, Tokushima University Graduate School, Japan, 3-18-15, Kuramoto-cho, Tokushima-shi, Tokushima 770-8503, Japan; Department of Radiation Oncology, Ise Red Cross Hospital, Japan, 1-471-2, Funae, Ise-shi, Mie 516-8512, Japan; Department of Radiation Oncology, Saitama Medical University, International Medical Center, Japan, 1397-1, Yamane, Hidaka-shi, Saitama 350-1298, Japan; Department of Radiology, Kyoto Prefectural University of Medicine, Japan, Kajii-cho, Kawaramachi-Hirokoji, Kamigyo-ku, Kyoto 602-8566, Japan; Department of Radiation Oncology, Juntendo University Graduate School of Medicine, Japan, 2-1-1, Hongo, Bunkyo-ku, Tokyo 113-8421, Japan; Department of Radiology, Kansai Medical University Medical Center, Japan 2-5-1, Shin-machi, Hirakata-shi, Osaka 573-1010, Japan; Diagnostic Radiology and Radiation Oncology, Graduate School of Medicine, Chiba University, Japan, 1-8-1, Inohara, Chuo-ku, Chiba-shi, Chiba 260-8670, Japan; Department of Radiological Technology, Teikyo University Graduate School of Medicine, Japan, 6-22, Misakimachi, Omuta-shi, Fukuoka 836-8505, Japan; Department of Radiation Oncology, Saitama Prefectural Cancer Center, Japan, 1696, Itai, Kumagaya-shi, Saitama 360-0197, Japan; Department of Radiology, Mie University Graduate School of Medicine, Japan, 2-174, Edobashi, Tsu-shi, Mie 5148-507, Japan; Radiation Therapy Center, Okinawa Chubu Hospital, Japan, 281, Miyasato, Uruma-shi, Okinawa 904-2293, Japan; Department of Radiation Oncology, Gunma University Graduate School of Medicine, Japan, 3-39-22, Showa-machi, Maebashi-shi, Gunma 371-8511, Japan; Department of Radiation Oncology, University of Tsukuba, Japan, 1-1-1, Tennoudai, Tsukuba-shi, Ibaraki 305-8575, Japan; Department of Radiology, Faculty of Medicine, University of Yamanashi, Japan, 4-4-37, Takeda, Kofu-shi, Yamanashi 400-8510, Japan

**Keywords:** brachytherapy, medical resources, national survey, patterns of care, resident education

## Abstract

This study aimed to assess the current state of brachytherapy (BT) resources, practices and resident education in Japan. A nationwide survey was undertaken encompassing 177 establishments facilitating BT in 2022. Questionnaires were disseminated to each BT center, and feedback through online channels or postal correspondence was obtained. The questionnaire response rate was 90% (159/177), and every prefecture had a response in at least one center. The number of centers in each prefecture ranged from 0.6 to 3.6 (median: 1.3) per million population. The annual number of patients in each center ranged from 0 to 272 (median: 31). While most prefectures provided intracavitary (IC) BT for gynecological cancers and interstitial (IS) BT for prostate cancer, only one-third of the prefectures provided IS BT for cancer sites other than the prostate. The institutional image-guided BT implementation rate was 71%. IC and IS BT was performed for 15.4% of IC BT cases of gynecological cancer. Only 47% of the BT training centers answered that they could provide adequate training in BT for residents. The most common reason for this finding was the insufficient number of patients in each center. The results show that, although BT has achieved uniformity in terms of facility penetration, new technologies are not yet widespread enough. Furthermore, IS BT, which requires advanced skills, is limited to a few BT centers, and considerable number of BT training centers do not have sufficient caseloads to provide the necessary experience for their residents.

## INTRODUCTION

Brachytherapy (BT) plays an essential role in radiation therapy (RT) of gynecological cancer. BT is one of the standard treatment methods for prostate cancer and is beginning to show efficacy as an accelerated partial breast irradiation (APBI) for early-stage breast cancer. Additionally, the application of BT could be expanded to anatomical loci amenable to an approach via interventional radiological methodologies. In these diseases, the radiation source is positioned in close proximity to or within the tumor itself, making it less susceptible to internal movement of the tumor. Additionally, BT allows for a reduction in the normal tissue dose because the dose decreases rapidly with increasing distance from the radiation source. However, when the precise localization of the radiation source within the vicinity of the tumor is unattainable, it leads to a decrease in tumor dosage and a concurrent escalation in the normal tissue dose. This culminates in a diminished efficacy of tumor control and an elevated incidence of adverse effects. In BT, the attainment of heightened accuracy is contingent on the proficiency and skill of radiation oncologists. Therefore, in addition to the health care economic situation, including insurance reimbursement, in which BT is included, the educational system of BT techniques also has a significant impact on the distribution of BT use. These circumstances vary across nations and regions, and consequently, the distribution of BT also varies by country, region and even institutions [1–13]. In Europe [3, 14], the number of patients treated with BT is increasing, with an average of >100 patients per center in the countries with the top one-third of the gross domestic product. In addition to gynecological and prostate cancers, which have been common targets, the use of APBI for breast cancer is increasing. In Latin America [6], the number of BT patients is in increasing tendency, with gynecological cancers being the most common. However, in the USA, the number of institutes that use external-beam RT for boost therapy for cervical cancer is increasing, and BT is on the decline [15]. One potential factor contributing to the diminution of BT within the USA is postulated to be a reduction in the residents’ experience with the practice of BT [16, 17]. In Korea [9], while the number of RT establishments is increasing, there is a concurrent decline in the number of facilities offering BT, which is predominantly attributed to financial difficulties.

To establish an appropriate system for the provision of BT throughout Japan, we must first understand the current status of BT. Additionally, a discussion is necessary to ascertain the appropriateness of the reimbursement framework allocated for BT within the confines of Japan’s universal health insurance system. In this study, we performed the first nationwide survey in Japan on the allocation of medical resources for BT, the number of patients treated by BT and residents’ educational status in relation to BT. Furthermore, we provide recommendations on the issues of BT in Japan, as delineated in the findings of this study.

## MATERIALS AND METHODS

The Japanese Group of BT/Japanese Society for Radiation Oncology (JGB/JASTRO) designed a questionnaire, which was mailed or emailed to all 177 BT centers in Japan between 1 June and 31 August 2022. The questionnaire consisted of questions regarding medical resources, collaborative efforts between centers, the number of patients per disease, the patient’s waiting status, the image-guided BT (IGBT) status and the educational attainment level of residents. The BT methods were classified as intracavitary (IC) BT, interstitial (IS) BT, IC + IS (IC/IS) BT and mold BT. The number of patients was defined as the total number of new and returning patients for whom BT was initiated between January and December 2021. Responses to the questionnaire were made on the internet or returned by mail to the JGB/JASTRO. A questionnaire sheet can be found in the Supplementary Appendix 1. The present study was approved by the Ethics Committee of Tokushima University Hospital (approval number: 4150-1).

## RESULTS

### Medical resources, patient’s waiting status and IGBT status

**Fig. 1:**
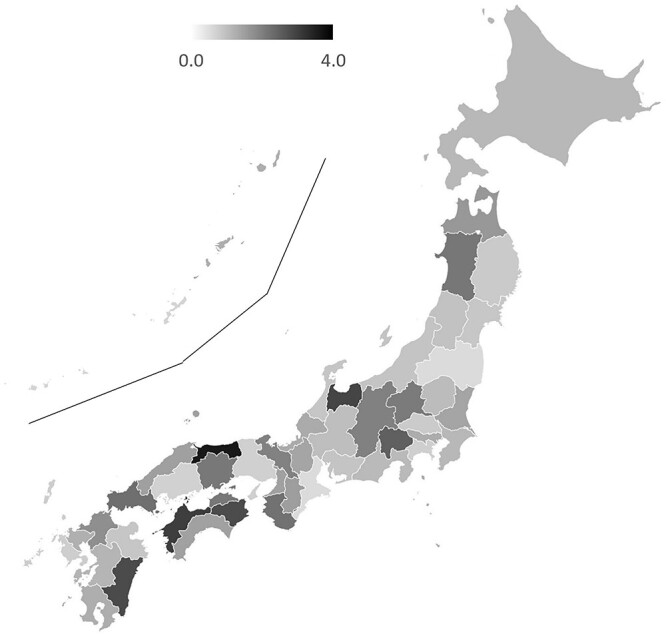
Number of BT centers per million population per prefecture. The islands of Kagoshima and Okinawa Prefectures are shown in the upper left of the map.

**Fig. 2:**
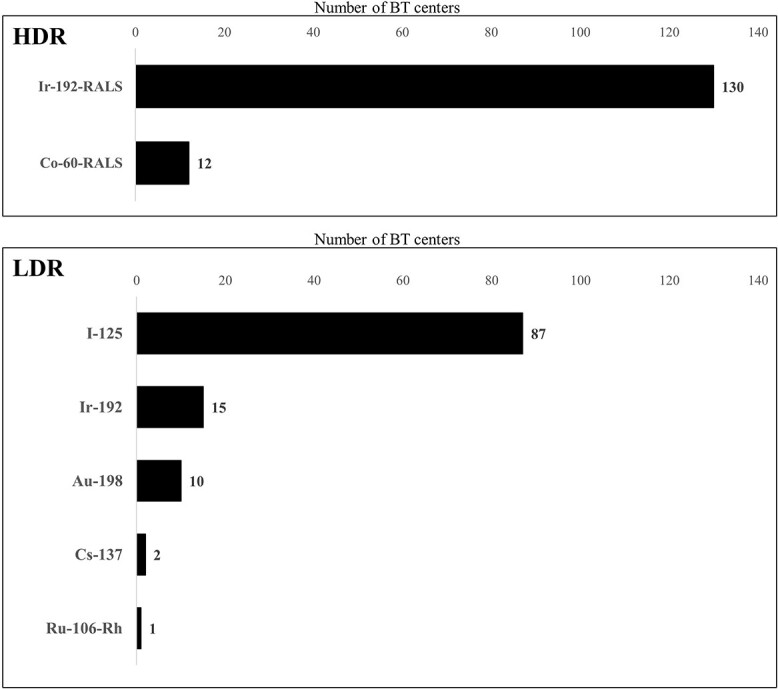
Types of remote after-loading systems (RALS) used for HDR BT and types of radioisotopes used for LDR BT. Ir = iridium, Co *=* cobalt, I *=* iodine, Au = aurum, Cs = cesium, Ru = ruthenium, Rh = rhodium.

**Fig. 3:**
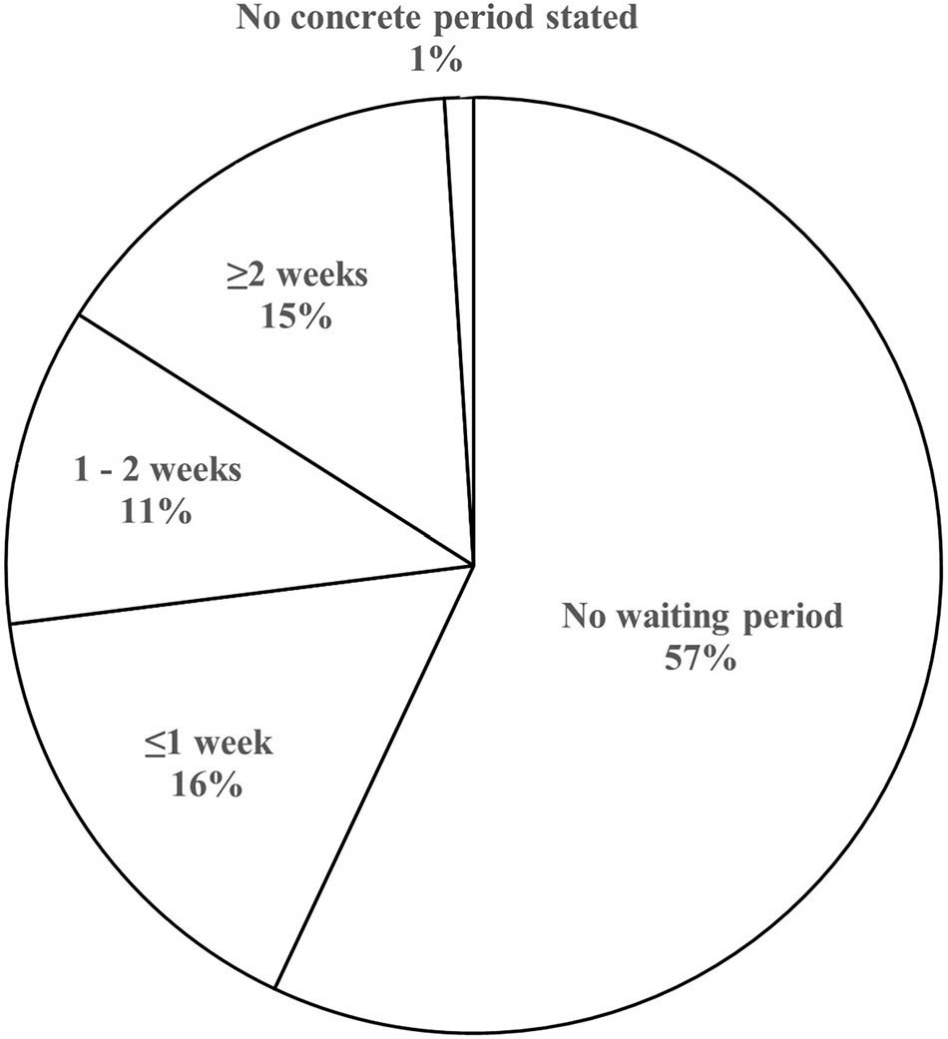
Average waiting period of patients for BT.

The survey response rate was 90% (159/177 BT centers). All prefectures had responses from at least one center. The number of BT centers in each prefecture ranged from 1 to 18 (median: 2), with per million population ranging from 0.6 to 3.6 (median: 1.3) (Fig. 1). The types of BT center and number of medical staff are shown in Table 1. The types of radioisotope used for high-dose rate (HDR) BT with a remote after-loading system and the radioisotope used for low-dose rate (LDR) BT are shown in Fig. 2. With regard to the capacity of the facility to accept patients, 50% of the centers answered that increasing the number of patients was possible, 43% answered that they were at their maximum capacity and 4% answered that they were already over capacity. The waiting periods of patients for BT are shown in Fig. 3. A total of 71% of the BT centers provided IGBT, and 89% (42/47) of the prefectures had BT centers that provided IGBT. The most common reason for not providing IGBT was inadequate facilities (37 centers), followed by a lack of staff (14 centers), knowledge and technical problems (12 centers), a lack of time (9 centers) and inadequate reimbursement (7 centers).

**Table 1:** BT centers and medical staff

Type of BT center	
University hospital or its branch	80 (50%)
Base hospital for cancer treatment other than a university hospital	57 (36%)
Cancer center	12 (7%)
Other general hospital	9 (6%)
Other	1 (1%)
Medical staff	
Number of radiation oncologists	
1	26 (16%)
2	30 (19%)
3	33 (21%)
4	25 (16%)
5	14 (9%)
≥6	31 (19%)
Average number of radiation oncologists and residents involved in one BT procedure	
1–1.5	63 (40%)
2	83 (52%)
3	11 (7%)
4	2 (1%)
Number of nurses in the RT Department^a^	
1–1.5	37 (23%)
2–2.5	56 (35%)
3	32 (20%)
4	15 (10%)
5–5.5	11 (7%)
≥6	8 (5%)
Average number of nurses involved in one BT procedure	
1–1.5	124 (78%)
2	33 (21%)
3	2 (1%)

^a^The number was set at 0.5 for part-time workers who work 2–3 days per week.

### Number of patients by prefecture and by BT center

**Fig. 4:**
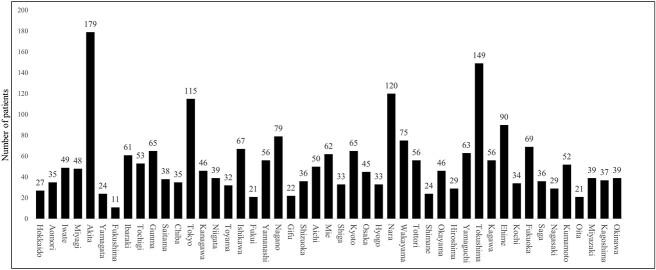
Total annual number of patients treated by BT in 2021 in each prefecture.

**Fig. 5:**
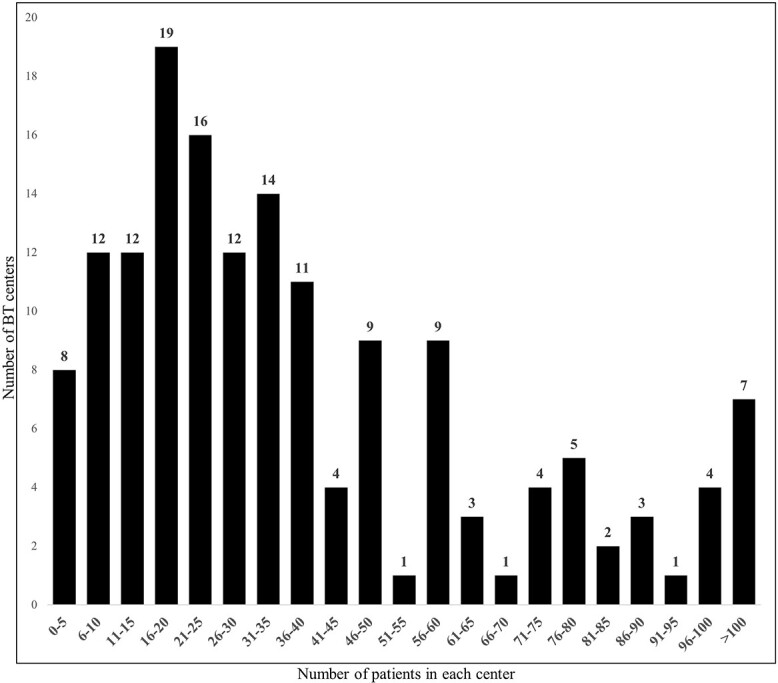
Distribution of the total annual number of patients treated by BT in 2021 in each center.

The total number of patients (new and returning patients) treated with BT in Japan between 1 January and 31 December 2021 was 6892. By prefecture, the number of patients ranged from 16 to 1617 (median: 85), and the per million population ranged from 11 to 179 (median: 46) (Fig. 4). The annual number of patients in each center ranged from 0 to 272 (median: 31), with eight (5%) centers admitting 5 or fewer patients each year (Fig. 5). Of these eight centers, four performed HDR only and four performed I-125 seed implantation for prostate cancer only. In addition, 87.5% (seven/eight) of these centers indicated that there were other BT centers in the same prefecture to which they could refer their patients.

### Number of patients by cancer site and treatment modality

**Fig. 6:**
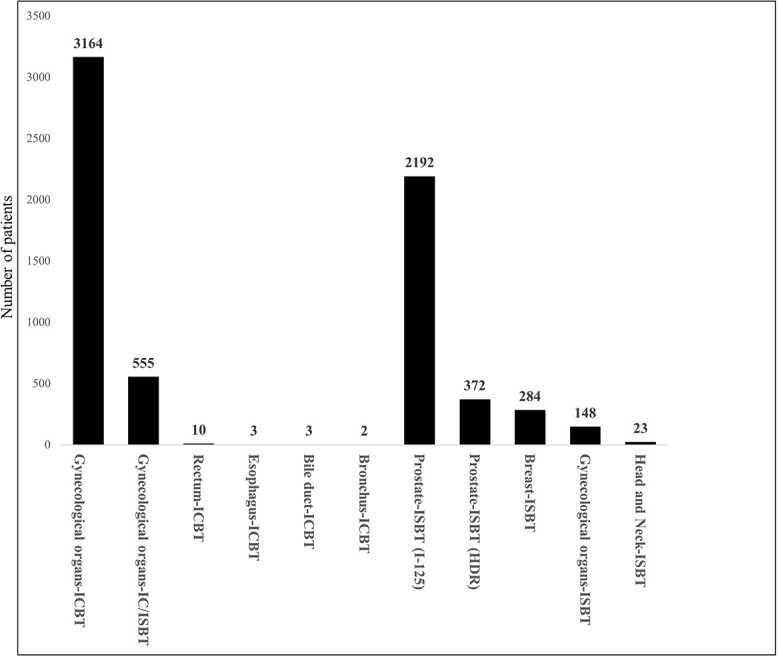
Number of patients treated by BT in 2021 according to the organ site and treatment modality.

The organ site and treatment modality with the largest number of patients was gynecological cancer treated with ICBT or IC/ISBT. A total of 3719 patients with gynecological cancer (cervical cancer: *n* = 2853, endometrial cancer: *n* = 187 and vaginal cancer: *n* = 124) were treated with ICBT. Additionally, 555 patients with gynecological cancer (cervical cancer: *n* = 518, endometrial cancer: *n* = 19 and vaginal cancer: *n* = 18) were treated by IC/ISBT. The second largest number was 2192 patients with I-125 seed implantation for prostate cancer, followed by 372 with HDR ISBT for prostate cancer, 284 with ISBT for breast cancer, 148 with ISBT for gynecological cancer (cervical cancer: *n* = 85, endometrial cancer: *n* = 28, vaginal cancer: *n* = 30 vulvar cancer: *n* = 5), 62 with LDR ISBT (Au-198 grain 56, Ir-192 5 and Cs-137 1) for head and neck cancers and 23 with HDR ISBT for head and neck cancers. ICBT for rectal, esophageal, biliary and bronchial cancers accounted for ≤10 patients (Fig. 6). Mold BT was delivered to 12 patients with oral cancer, 6 patients with skin malignancies, 1 patients with a keloid and 1 patients with breast cancer. ICBT for gynecological cancer, which was the most common reason for BT, was performed in all prefectures. The number of patients per million population per prefecture ranged from 9 to 61 (median: 29). The number of patients per million population per prefecture of ISBT for prostate cancer (I-125 seed implantation and HDR ISBT), which was the second most common reason for BT, ranged from 0 to 127 cases (median: 14 patients), with no patients treated in 5 prefectures. By contrast, only 35% (20/47) of the prefectures had BT centers that provided ISBT for cancers other than prostate cancer.

### Resident education of BT

**Fig. 7:**
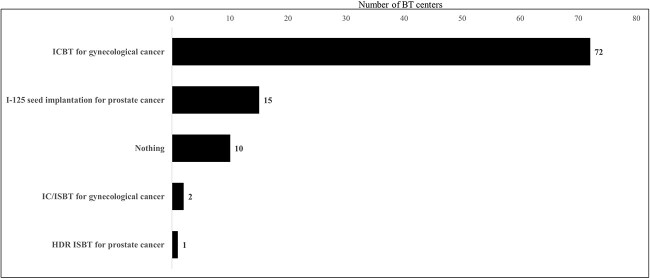
The diseases and treatment techniques that could be entrusted to be performed alone after the completion of training.

A total of 60% (95/159) of the BT centers indicated that they offered educational program in BT for resident. Forty-four prefectures had BT centers available for resident training in BT (Table 2). With regard to the total number of residents by prefecture, Tokyo, Kanagawa, Aichi and Osaka prefectures had more than five residents, while most other prefectures had less than two. The primary training method (multiple responses allowed) for BT in 95 training BT centers was as an assistant of a radiation oncologist at 75 centers, followed by resident-initiated delivery at 52 centers, observation at 40 centers and lectures at 15 centers. The diseases and treatment techniques that could be entrusted to be performed alone after the completion of training at the 95 BT training centers are shown in Fig. 7. A total of 47.4% (45/95) of the BT training centers responded that they could provide adequate training in BT at their centers, 29.5% (28/95) did not think they could and 23.2% (22/95) were undecided. When respondents did not believe that adequate BT training was possible or were undecided, the reasons provided were an insufficient number of patients (29 centers), insufficient instructors (20 centers), problems securing resident time (12 centers) and insufficient equipment (10 centers). To enhance the education of BT, the following opinions were provided: enhancement of medical staff and equipment through the centralization of the BT centers and an increase in the number of the patients; collaboration among BT centers to enable training at high-volume centers; establishment of educational programs and provision of educational content, such as e-learning and hands-on seminars by the JASTRO and training of BT supervisors.

**Table 2:** Resident education of BT

Number of residents in 2021 in 95 BT training centers	
0	16 (16.8%)
1	37 (38.9%)
2	16 (16.8%)
3	13 (13.7%)
4	5 (5.3%)
≥5	8 (8.4%)
Average number of patients allocated for one resident instruction annually in 79 centers that offered educational programs in BT in 2021	
Gynecological cancer	
0	8 (10.1%)
1–5	39 (49.4%)
6–10	18 (22.8%)
≥11	14 (17.7%)
Prostate cancer	
0	41 (51.9%)
1–5	23 (29.1%)
6–10	7 (8.9%)
≥11	8 (10.1%)
Head and neck cancer	
0	73 (92.4%)
1–5	4 (5.1%)
≥6	2 (2.5%)

## DISCUSSION

BT centers were established within each prefecture. Therefore, the entirety of the prefectures was encompassed within the purview of this study. The present survey showed a good response rate of 90%, and feedback was provided from university-affiliated medical institutions and non-university cancer treatment-providing hospitals. This comprehensive spectrum also encompassed cancer centers spanning all prefectures. Therefore, this survey included data that accurately represent the prevailing landscape of BT within Japan.

The number of BT centers per million population, allocated across each prefecture within Japan, ranged from 0.6 to 3.6 (median: 1.3). Our survey suggested that the allocation of BT facilities covered all regions of Japan. All centers were staffed with at least one physician and one nurse specializing in BT, and at least 10 hours each week were distributed to BT practice (detailed data on ICBT for cervical cancer have already been reported by Toita *et al*. in 2018 [18]). Regarding the temporal lag for patients awaiting BT, ~43% of the BT centers had patient wait times, with ~15% experiencing delays of ≥2 weeks. The current study did not compile data regarding the wait duration based on targeted disease and therapeutic modalities. However, the outcomes may mirror the interval for ISBT in cases of prostate cancer, which is frequently scheduled after preliminary hormone therapy or external-beam radiation treatment. Notably, only 4% of the facilities reported exceeding capacity in accommodating BT, which suggests a preponderance of supply in the equilibrium between provisioning and demand for BT facilities. The use of BT in Japan appears to have achieved sufficient equalization in terms of the number of facilities and a minimum level of medical staffing. While the number of patients in each center varied, the median number of patients per year was 31, which is much less than that reported in other countries [3, 6, 7, 11]. In particular, 5% of BT centers reported notably limited patient caseloads (<five patients/year). A substantial proportion of these establishments indicated the presence of proximate facilities to which they can refer patients. Consequently, these BT centers should consider strategies for centralization, referring patients to nearby centers. Nonetheless, this endeavor must also encompass an assessment of the socio-contextual milieu in each locality, including the patient’s access to referral BT centers.

The IGBT implementation rate in Japan has increased from 48% in 2016 [18] to 71% in 2021, but it is still inadequate compared with Europe [20] and North America [21, 22]. The main reason for this is that IGBT require additional facilities. The IC/IS implementation rate for gynecological cancer is also only 15.4% of the total ICBT (ICBT + IC/ISBT). The adoption of such novel technologies necessitates a commensurate allocation of capital resources, and suboptimal insurance reimbursement can constitute a formidable impediment to the diffusion of these technologies. Currently in Japan, there remains no possibility of insurance reimbursement for needles used in ISBT, and as the quantity of needles escalates progressively diminishes the treatment’s financial viability progressively diminishes. Furthermore, ISBT or IC/ISBT represents a notably more invasive treatment than ICBT, where procedural sedation and analgesia are indispensable, and allocating an adequate complement of medical staff is imperative. The enhancement of insurance reimbursement has the potential to mitigate the impediments associated with achieving parity for emerging technologies throughout the nation.

The largest number of patients according to the tumor site and treatment modality was for ICBT for gynecological cancer, followed by ISBT for prostate cancer, ISBT for breast cancer and ISBT for gynecological cancer. The numbers of patients who received ISBT for head and neck cancers and the numbers who received ICBT for other than gynecological cancer were small. These results are similar to those reported from other countries [3, 6, 7, 9, 11]. Gynecological ICBT was performed in all prefectures. ISBT for prostate cancer, which was the second most common type of BT, was also performed in almost all prefectures (42/47 prefectures). These findings suggest that equalization of treatment in these two tumor sites and modalities in the centers has been achieved. However, only approximately one-third of the prefectures in Japan provide ISBT for cancer other than prostate cancer, which requires advanced techniques. ISBT is one of the recommended treatments in APBI for breast cancer [19], and it is effective for gynecological tumors that cannot be adequately treated with ICBT or IC/ISBT, such as large or recurrent tumors. To optimize the provision of BT, we propose that an establishment dedicated to ISBT, catering to diverse cancer sites other than the prostate, should be established within the designated areas across prefectural borders to integrate services.

Only 47% of the BT training centers answered that they were able to provide adequate training in BT for residents at their institutions. Additionally, except for ICBT for gynecologic cancer, the proportion of residents deemed capable of performing BT procedure alone post-training was limited. The most common reason for this finding was the insufficient number of patients in each center. A survey conducted in the USA [16, 17] and Canada [23] on residents’ education in BT reported that, although centers recognize the importance of education, they are unable to provide adequate training because they cannot obtain enough patients other than those with gynecological ICBT. Residents need to experience a sufficient number of cases during their training period to gain confidence in their ability to perform BT on their own after training. Our survey showed that equalization of the number of facilities and equipment has been achieved. However, in Japan, there are many centers with a small number of cases in each center. Therefore, an educational system led by the JASTRO which includes inter-institutional collaboration to enable training at high-volume centers and more practical seminars needs to be established so that residents can obtain technical practice. This survey additionally underscored the shortage of nurses available to provide BT. To provide high-quality BT, it is imperative not only to educate physicians but also to augment both the quantity and proficiency of nursing staff. In 2022, the JASTRO commenced organizing periodic hands-on workshops [24]. BT necessitates hands-on instruction in procedural methodologies, which means that the spectrum of practical training opportunities, transcending the mere confines of didactic lectures, needs to be expanded.

## CONCLUSIONS

BT has achieved uniformity in terms of facility penetration and is readily available in all areas of the country, but the number of patients and the BT procedures offered vary considerably between centers. New technologies, such as IGBT and IC/ISBT, are not yet widespread enough. Furthermore, ISBT for cancers other than prostate cancer, which requires advanced skills, is limited to a few BT centers, and a considerable number of BT centers do not have sufficient caseloads to provide the necessary experience for their residents.

## Supplementary Material

Appendix_1_Questionnaire_sheet_rrad099
