# Supplementary material for: Patterns of care for brachytherapy in Japan
Source: J Radiat Res. 2023 Dec 26;65(2):168–76. doi: 10.1093/jrr/rrad099 (PMC10959427; doi:10.1093/jrr/rrad099)
Supplement: Appendix_1_Questionnaire_sheet_rrad099 [file appendix_1_questionnaire_sheet_rrad099.pdf]

No.	Questionnaire	Choices
1	Facility name	
2	Location of the facility (prefecture)	
3	Type of facility	University hospital or its branch・Base hospital for cancer treatment other than a university hospital・Cancer center・Other general hospital・Other
4	Name of respondent	
5	Please select the remote afterloading system (RALS) you use for high-dose-rate (HDR) brachytherapy. (Multiple choices allowed)	MicroSelectron HDR・Flexitron HDR・Varisource・MutltiSource (^{192}Ir-RALS)・MutltiSource (^{60}Co-RALS)・BRAVOS・SagiNova (^{192}Ir-RALS)・SagiNova (^{60}Co-RALS)
6	Please select the low-dose-rate (LDR) brachytherapy system/ source you use. (Multiple choices allowed)	^{125}I permanent implantation system・^{192}Ir(LDR)・^{137}Cs (LDR)・^{198}Au grain・^{106}Ru-Rh・^{90}Sr-Y
Questions 7-10 relate to the number of healthcare workers; please state the number as at 1 April 2022.		
7	Number of radiation oncologist (full-time + part-time)	
8	Number of nurses in radiation oncology department. The number was set at 0.5 for part-time workers who work 2 to 3 days per week.	
9	Average number of radiation oncologists and residents involved in one brachytherapy procedur.	
10	Average number of nurses involved in one brachytherapy procedure.	
11	Average time spent on brachytherapy as a team of radiation oncologists and nurses per week. (hour)	
12	Is it possible to increase the number of brachytherapy from the current level at your institution?	Possible・At our maximum capacity・Already over capacity・Unknown
13	Patient waiting periods for brachytherapy.	No waiting period・≤1 week・1 to 2 weeks・≥2 weeks
14	Do you provide image-guided brachytherapy (IGBT)? (Please indicate the status as at 1 April 2022)	Yes・No
15	Reasons for not performing IGBT.	Knowledge and technical problems・Lack of staff・ Inadequate facilities・Lack of time・No need (2D is sufficient)・Inadequate reimbursement ・Other (If other, please state in the next.)
16	Please state if the reason is other.	
17	Do you accept patients for brachytherapy from other facilities? (as of 1 April 2022)	Yes・No
18	Number of other facilities from where you acceptes patients for BT. (One year, Jan-Dec 2021)	
19	Location of the other facility from where you accepted the patients. (Multiple choices allowed)	Same prefecture・Adjacent prefecture・Distant prefecture
20	Number of patients accepted from other facilities. (One year, Jan-Dec 2021)	
21	If you do not accept patients from other facilities, what are the reasons? (Multiple choices allowed)	No referrals/ Referrals but cannot afford to accept/ Other (If other, please state in the next.)
22	Please state if the reason is other.	
23	Are there neighbouring facilities (in the same or neighbouring prefecture) to which you can refer if you need brachytherapy that cannot be performed at your institution?	Yes・No
In the following questions 24-56, please state the number of patients (new patients + return patients) from 1 January to 31 December 2021.		

Intracavitary brachytherapy (ICBT) using HDR-RALS		
24	Esophagus	
25	Bile duct	
26	Bronchus	
27	Rectum	
28	Uterine cervix	
29	Uterine body	
30	Vagina	
31	List other diseases (if more than one, please list them all here).	
32	Number of patients with other diseases. (Please provide the total number even if there is more than one disease.)	
Intracavitary and Interstital brachytherapy (IC/ISBT) using HDR-RALS		
33	Uterine cervix	
34	Uterine body	
35	Vagina	
Interstital brachytherapy (ISBT) using HDR-RALS		
36	Head and neck	
37	Breast（SAVI catheter)	
38	Breast (Multicatheter）	
39	Uterine cervix	
40	Uterine body	
41	Vagina	
42	Prostate	
43	List other diseases. (If more than one, please list them all here).	
44	Number of patients with other diseases. (Please provide the total number even if there is more than one disease.)	
Mold brachytherapy using HDR-RALS		
45	Oral	
46	Skin	
47	Keroid	
48	List other diseases. (If more than one, please list them all here.)	
49	Number of patients with other diseases (Please provide the total number even if there is more than one disease.)	
LDR brachytherapy		
50	^{125}I-125 seed implantation for prostate cancer	
	LDR using ^{192}Ir	
51	List disease of LDR using ^{192}Ir. (If more than one, please list them all here.)	
52	Number of patients with LDR using ^{192}Ir. (Please provide the total number even if there is more than one disease)	

	LDR using ^{137}Cs	
53	List disease of LDR using ^{137}Cs. (If more than one, please list them all here.)	
54	Number of patients with LDR using ^{137}Cs (Please provide the total number even if there is more than one disease.)	
	LDR using ^{198}Au grain	
55	List disease of LDR using ^{198}Au grain. (If more than one, please list them all here.)	
56	Number of patients with LDR using ^{198}Au grain (Please provide the total number even if there is more than one disease.)	
57	Can you provide training in brachytherapy in your center?	Yes・No
58	Number of residents per center trained in BT in 2021	
59	Number of patients for training per resident in 2021：Gynecological cancer	
60	Number of patients for training per resident in 2021：Prostate cancer	
61	Number of patients for training per resident in 2021：Head and neck cancer	
62	The primary training method for BT. (Multiple choices allowed)	Resident-initiated delivery・Assistant of a radiation oncologist・Observation・Lectures
63	The diseases and treatment techniques that could be entrusted to be performed alone after the completion of training (Based on the opinions of the residents)	ICBT for gynecological cancer・IC/ISBT for gynecological cancer・ISBT for gynecological cancer・ ^{125}I seed implantation for prostate cancer・ISBT for prostate cancer・Nothing・Other (If other, please state in the next.)
64	List all diseases and treatments in other cases.	
65	Do you think adequate brachytherapy training is available at your institution?	Possible・Impossible・Undecided
66	If you do not think adequate brachytherapy training is available or are undecided, what are the reasons? (Multiple choices allowed)	Insufficient instructors・Insufficient equipment・Problems securing resident time・Insufficient number of patients・Other (If other, please state in the next.)
67	Please state if the reason is other.	
68	Your opinions on what needs to be done to improve brachytherapy education (Free text)	
